# Discovering Skin Anti-Aging Potentials of the Most Abundant Flavone Phytochemical Compound Reported in Siam Violet Pearl, a Medicinal Plant from Thailand by In Silico and In Vitro Assessments

**DOI:** 10.3390/antiox14030272

**Published:** 2025-02-26

**Authors:** Chaiyawat Aonsri, Sompop Kuljarusnont, Duangjai Tungmunnithum

**Affiliations:** 1 Department of Pharmaceutical Chemistry, Faculty of Pharmacy, Mahidol University, Bangkok 10400, Thailand; chaiyawat.aon@mahidol.ac.th; 2 Unit of Compounds Library for Drug Discovery, Mahidol University, Bangkok 10400, Thailand; 3 Department of Obstetrics and Gynecology, Faculty of Medicine Siriraj Hospital, Mahidol University, Bangkok 10700, Thailand; sompop.kul@mahidol.edu; 4 Department of Pharmaceutical Botany, Faculty of Pharmacy, Mahidol University, Bangkok 10400, Thailand; 5 Le Studium Institute for Advanced Studies, 1 Rue Dupanloup, 45000 Orléans, France

**Keywords:** flavone, *Monochoria angustifolia*, flavonoids, medicinal plants, anti-aging, molecular modeling, pharmacological activity, medical benefits

## Abstract

Currently, nutraceuticals and functional food/cosmeceutical sectors are seeking natural molecules to develop various types of phytopharmaceutical products. Flavonoids have been reported in antioxidant and many medical/pharmacological activities. *Monochoria angustifolia* or Siam violet pearl medicinal plant is the newest species of the genus *Monochoria* C. Presl, which have long been consumed as food and herbal medicines. Though previous work showed that apigenin-7-*O*-glucoside is the most abundant antioxidant phytochemical found in this medicinal plant, the report on anti-aging activity is still lacking and needs to be filled in. The objective of this work is to explore anti-aging capacities of the most abundant antioxidant phytochemical reported in this plant using both in silico and in vitro assessments. In addition, pharmacokinetic properties were predicted. Interestingly, the results from both in silico and in vitro analysis showed a similar trend that apigenin-7-*O*-glucoside is a potential anti-aging agent against three enzymes. The pharmacokinetic properties, such as adsorption, distribution, metabolism, excretion and toxicity (ADMET), of this compound are also provided in this work. The current study is also the first report on anti-aging properties of this Thai medicinal plant. However, the safety and efficacy of future developed products from this compound and clinical study should be determined in the future.

## 1. Introduction

Flavonoids are one of the large classes of phytochemical compounds synthesized by several plants species which have been reported as having various medical/pharmacological benefits [1,2,3,4,5,6,7,8,9,10,11,12,13,14], especially anti-aging effects and in the treatment of degenerative diseases [5,15,16,17,18,19,20,21,22,23,24,25,26,27]. The antioxidant potential is one of the key pharmacological activities of flavonoids such as flavones, flavanones, flavonols, flavanonol, isoflavones, aurones, chalcones and anthocyanin, which has been extensively reported globally [1,2,3,4,5,6,7,8,9,11,12,13,14,15,16,17,18,19,20,21,22,24,25,26,27]. Currently, customers’ preference for natural products is sharply increasing. Therefore, nutraceuticals, functional food, cosmetics, cosmeceutical and other phytopharmaceutical industries are currently focusing on the natural bioactive molecules for various product developments.

**Figure 1:**
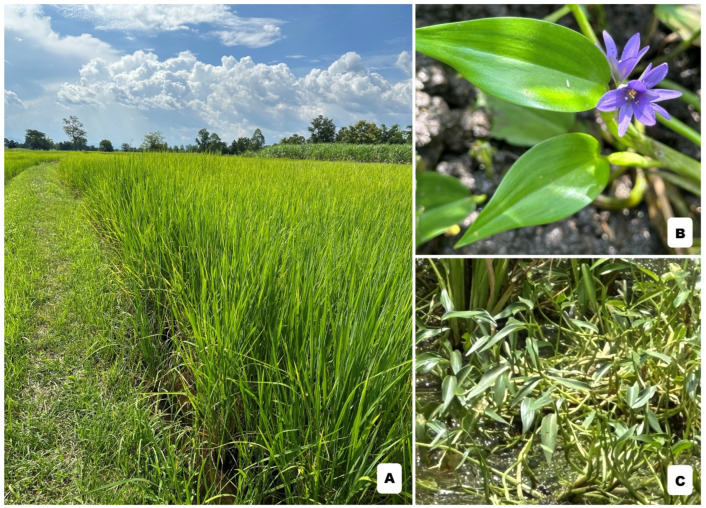
*M. angustifolia*: (**A**) rice field, its natural habitat in Thailand; (**B**) floral part; (**C**) vegetative part. All photos were taken in Thailand by Assoc. Prof. Dr. Duangjai Tungmunnithum.

*Monochoria angustifolia* (G. X. Wang) Boonkerd & Tungmunnithum or the so-called Siam violet pearl is a newly discovered medicinal plant species from Thailand (Figure 1) [28,29,30]. This medicinal plant is also known by various vernacular names, i.e., Phak Lin, Phak Xi Hin or Khimuk Si Muang Haeng Siam, depending on the region/locality [28,29,30,31,32]. *M. angustifolia* is a species member of the genus *Monochoria* C. Presl (Pontederiaceae family) that has long been used in cooking as a vegetable, cosmetics and many herbal medicinal remedies [24,28,31]. Our previous study determined flavonoid phytochemical profiles of 25 *M. angustifolia* populations from their natural habitat, covering all of the floristic regions in Thailand, and also determined their antioxidant potential using five in vitro antioxidant assays, i.e., ABTS (2,2-azinobis(3-ethylbenzthiazoline-6-sulphonic acid)), FRAP (ferric reducing antioxidant power), ORAC (oxygen radical absorbance capacity assay) DPPH (2,2-diphenyl-1-picrylhydrazyl) and CUPRAC (cupric reducing antioxidant capacity) covering different antioxidant mechanisms as well as a cellular antioxidant assay using a yeast model [24]. The results from those various antioxidant analyses in the previous research showed that *M. angustifolia* extracts exhibited the antioxidant action via a hydrogen atom transfer antioxidant mechanism [24].

In addition, the results from our previous research work showed that Apigenin-7-*O*-glucoside, a flavone compound, is the most abundant antioxidant flavonoid occurring in this medicinal plant species (36.84 ± 0.49 mg/g DW) [24] and provided high antioxidant effects, determined by FRAP (503.66 ± 84.40 μmol TEAC), CUPRAC (225.45 ± 42.7 μmol TEAC), ABTS (187.95 ± 1.78 μmol TEAC), DPPH (326.19 ± 6.25 μmol TEAC) and ORAC (400.79 ± 40.41 μmol TEAC) [24]. However, there are no reports on anti-aging activity, which is an interesting pharmacological activity that can be applied in various nutraceuticals, functional foods, cosmetic/cosmeceutical and other phytopharmaceutical product development. In addition, molecular docking is a powerful computer-aided drug design (CADD) methodology that plays a crucial role in the drug discovery process, enabling researchers to predict potential drug candidates through computational algorithms. This method visualizes molecular orientation, bond distances and binding interactions between three-dimensional protein structures and chemical candidates, which serve as critical decisional factors [33,34]. CADD is also recognized as a green approach, as it reduces the chemical and biological waste commonly produced in experimental workflows [35] and provides additional benefits, such as rapid analysis, cost-efficiency and saving time by narrowing the selection of potential drug candidates [36]. Open access webservers are widely used to estimate pharmacokinetic properties—adsorption, distribution, metabolism, excretion and toxicity (ADMET)—based on quantitative structure–activity relationship (QSAR) databases [37,38] or machine learning [39], in order to gain the fundamental information to see the overview of the pharmacokinetic properties of the targeted compounds.

Thus, the current study aims to investigate the skin anti-aging potentials of the most abundant antioxidant phytochemical compounds, apigenin-7-*O*-glucoside, reported in Siam violet pearl medicinal plant (*M. angustifolia*) by using both in silico molecular docking and the three different in vitro enzymatic assessments. In addition, the pharmacokinetic properties, such as adsorption, distribution, metabolism, excretion and toxicity, of this compound were also provided as an overview of its pharmacokinetic properties.

## 2. Materials and Methods

### 2.1. Molecular Docking

Apigenin-7-*O*-glucoside (Api-7-*O*-Glc, PubChem CID: 12304093), a major flavonoid glucoside isolated from *M. angustifolia* [24], along with three positive control compounds—1,10-phenanthroline (PubChem CID: 1318), oleanolic acid (PubChem CID: 10494) and kojic acid (PubChem CID: 3840)—were modeled as three-dimensional structures using Chem3D Pro 12.0 software (PerkinElmer Inc., Cambridge, MA, USA). The 3D structures were then optimized to achieve minimized energy conformations using MM2 minimization [40] and CHARMm force field [41,42]. These constructed molecules were docked into three anti-aging enzymes: collagenase G from *Clostridium histolyticum* (PDB ID: 7ZBV, resolution 1.95 Å) [43], porcine pancreatic elastase (PDB ID: 1BRU, resolution 2.30 Å) [44] and tyrosinase from *Agaricus bisporus* (PDB ID: 2Y9X, resolution 2.78 Å) [45]. The crystal structures of these enzymes were downloaded from the Protein Data Bank (PDB, https://www.rcsb.org/, accessed on 14 September 2024) [46,47] and prepared by adding hydrogen atoms and removing co-crystalized ligands, cofactors and water molecules prior to docking. The GOLD software suit version 5.7.1 (Genetic Optimization for Ligand Docking, CCDC, Cambridge, UK) [48] was used to investigate the potential protein–ligand interactions within 12 angstrom (Å) radius, running 100 docking iterations per molecule with both GoldScore and ChemScore scoring functions at 100% efficiency. Api-7-*O*-Glc and three positive controls were docked into the central coordinates (x, y, z) of each enzyme’s binding site, based on the center of the co-crystalized ligand within the enzymatic crystal structures. The central coordinates for collagenase G, elastase and tyrosinase were (10.86, −1.76, 12.65), (23.20, 47.66, 17.09) and (−10.02, −28.82, −43.59), respectively. Discovery Studio Visualizer V.24.1.0 (BIOVIA, San Diego, CA, USA) [49] was used to visualize the three-dimensional interaction graphics of the docking results.

### 2.2. Prediction of Pharmacokinetic Properties and Toxicities

The pharmacokinetic properties and toxicity profiles of Api-7-*O*-Glc were predicted using online webservers. SwissADME (http://www.swissadme.ch/, accessed on 14 September 2024) [50] and the pKCSM (https://biosig.lab.uq.edu.au/pkcsm/, accessed on 14 September 2024) [51] webservers were utilized to evaluate partition coefficient (Log P), solubility (Log S), water solubility and skin permeability (Log Kp). The ProTox 3.0 (https://tox.charite.de/protox3/, accessed on 14 September 2024) [52] webserver was used to assess skin sensitization, hepatotoxicity, carcinogenicity and toxicity classification.

### 2.3. In Vitro Enzymatic Anti-Aging Assays

#### 2.3.1. Chemical Reagents

All of the reagents were purchased from Sigma-Aldrich (St. Louis, MO, USA). All of the standards are HPLC grade, with a purity of ≥98%, obtained from Extrasynthese (Genay Cedex, France). All solvents are analytical grade from Thermo Scientific (Waltham, MA, USA).

#### 2.3.2. Determination of the Anti-Collagenase Assay

The collagenase from clostridium histolyticum (Sigma Aldrich) is used for this experiment, and collagenase activity was determined by the spectrophotometer (Shimadzu, Kyoto, Japan) using its substrate, *N*-[3-(2-furyl)acryloyl]-Leu-Gly-Pro-Ala (FALGPA; Sigma Aldrich), according to the protocol suggested by the previous report of Wittenauer et al. [53] with minor modifications. The absorbance was measured to monitor the decrease in the FALGPA at 335 nm over a 20 min period using a microplate reader (BMG labtech, Victoria, Australia). The measurements were conducted in triplicate; the anti-collagenase activity was revealed as % inhibition relative to the control for every sample. The concentrations of standard compound were 100 µM. The collagenase’s specific inhibitor such as 1,10-phenantroline (100 μM) was used as the positive control of this enzyme. The % inhibition of collagenase was calculated by comparing with the initial slope during the first 10 min to the control using the following equation:

$$\%\;\mathrm{inhibition}\;\mathrm{of}\;\mathrm{collagenase}=\frac{{\mathrm{Slope}}_{\mathrm{control}\;}-{\mathrm{Slope}}_{\mathrm{sample}}}{{\mathrm{Slope}}_{\mathrm{control}}}\times 100$$

#### 2.3.3. Determination of the Anti-Elastase Assay

The elastase assay was determined by using porcine pancreatic elastase (Sigma Aldrich) and elastase’s activity was investigated by the spectrophotometer (Shimadzu, Japan). The *N*-Succ-Ala-Ala-Alap-nitroanilide (AAAVPN; Sigma Aldrich) was used as its substrate and *p*-nitroaniline’s release was followed at 410 nm by using the microplate reader (BMG labtech, Victoria, Australia) adapted based on the method that was previously reported by Wittenauer et al. [53] with minor modifications. The measurements were conducted in triplicate; the anti-elastase activity was presented in the form of a percentage of inhibition relative to the control. The concentration of standard compound was 100 µM. The 10 μM of oleanolic acid was employed as a positive control of this enzyme. The calculation of % inhibition was performed as described previously in the case of collagenase assay using the following equation:

$$\%\;\mathrm{inhibition}\;\mathrm{of}\;\mathrm{elastase}=\frac{{\mathrm{Slope}}_{\mathrm{control}\;}-{\mathrm{Slope}}_{\mathrm{sample}}}{{\mathrm{Slope}}_{\mathrm{control}}}\times 100$$

#### 2.3.4. Determination of the Anti-Tyrosinase Assay

The tyrosinase assay was performed following the previous method described by Chai et al. [54] with minor modifications. In brief, the 5 mM L-DOPA (3,4-dihydroxy-L-phenylalanine; Sigma Aldrich) was used as the substrate, and then mixed in sodium phosphate buffer (50 mM, pH 6.8) with 10 μL of the sample. After that, 0.2 mg/mL of the mushroom tyrosinase solution (Sigma Aldrich) was added to the mixture, so as to reach the final volume at 200 μL. The reaction was then detected by using the microplate reader (BMG labtech, Victoria, Australia) at 475 nm. The concentrations of standard compound were 100 µM. The tyrosinase inhibitory effect was presented as the % inhibition relative to the control. The 10 μM of Kojic acid was used as the positive control of this enzyme. The calculation of % inhibition was performed as described previously in the case of collagenase assay using the following equation:

$$\%\;\mathrm{inhibition}\;\mathrm{of}\;\mathrm{tyrosinase}=\frac{{\mathrm{Slope}}_{\mathrm{control}\;}-{\mathrm{Slope}}_{\mathrm{sample}}}{{\mathrm{Slope}}_{\mathrm{control}}}\times 100$$

### 2.4. Statistical Analysis

The data contained at least 3 independent replicates, and the data were shown as means and standard deviations. The statistical analysis was determined using Student’s *t*-test for the statistical comparative analysis. Significant differences at *p* < 0.05 and 0.01 were represented by * and **. Different letters were employed to indicate significant differences at *p* < 0.05.

## 3. Results and Discussion

### 3.1. Binding Interactions Predicted by Molecular Docking

**Figure 2:**
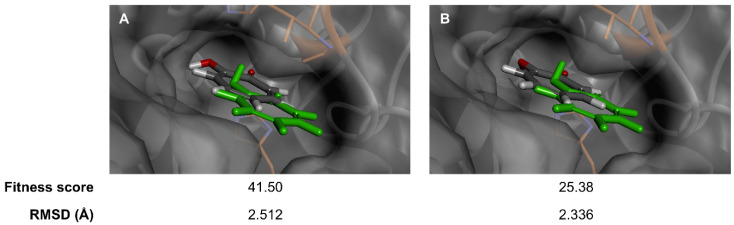
Validated docking studies of 2-hydroxycyclohepta-2,4,6-trien-1-one within binding site of tyrosinase enzyme, via (**A**) GoldScore and (**B**) ChemScore scoring function, in relation to co-crystallized ligand (green).

Flavonoids and their glycosides are valuable plant metabolites and have served extensively as active ingredients in medicines [55], supplements [56] and cosmetics [57], especially for their potential anti-aging candidates [58,59]. Api-7-*O*-Glc, a major component in a traditional lotus, namely *M. angustifolia* [24], found in Thailand, was first used to analyze the anti-aging activity through molecular docking studies, alongside in vitro assays, targeting three skin-aging-related enzymes, including collagenase, elastase and tyrosinase. The reliability of the scoring functions, GoldScore and ChemScore, and reproducibility of binding interactions were validated by self-docking 2-hydroxycyclohepta-2,4,6-trien-1-one, the co-crystallized ligand, into the binding site of tyrosinase. Due to coordination with the copper (II) ion, the postures computed by GoldScore and ChemScore showed minor deviations from the crystal structure (Figure 2), with a root-mean square deviation (RMSD) value of 2.512 and 2.336 Å, respectively. These RMSD values, both less than 3 Å, indicated acceptable and reliable docking results [60,61]. GoldScore was selected for further docking studies based on its higher fitness score relative to ChemScore.

The fitness scores of Api-7-*O*-Glc and positive controls are presented in Table 1, based on conformations that fulfilled three critical criteria: (i) precise alignment within the enzyme’s binding cavity, (ii) avoidance of repulsive interactions and (iii) a favorable overlay with the co-crystallized ligand. The docked molecule that maintains an optimal distance and exhibits a strong binding affinity to the enzyme’s binding cavity residues generally tends to achieve a higher fitness score [62]. Api-7-*O*-Glc showed potential as an anti-aging inhibitor, with its fitness scores 1.15 to 1.62 times higher than those of positive controls across all tested enzymes. Notably, Api-7-*O*-Glc exhibited the highest fitness score for collagenase in comparison with other two skin-aging-related enzymes, elastase and tyrosinase. This suggested that Api-7-*O*-Glc could be a promising candidate for targeting the skin collagenase enzyme in anti-aging treatments. Moreover, the fitness scores of other flavonoids isolated from *Monochoria angustifolia* (G. X. Wang) Boonkerd & Tungmunnithum are also docked and reported in Table S1.

**Figure 3:**
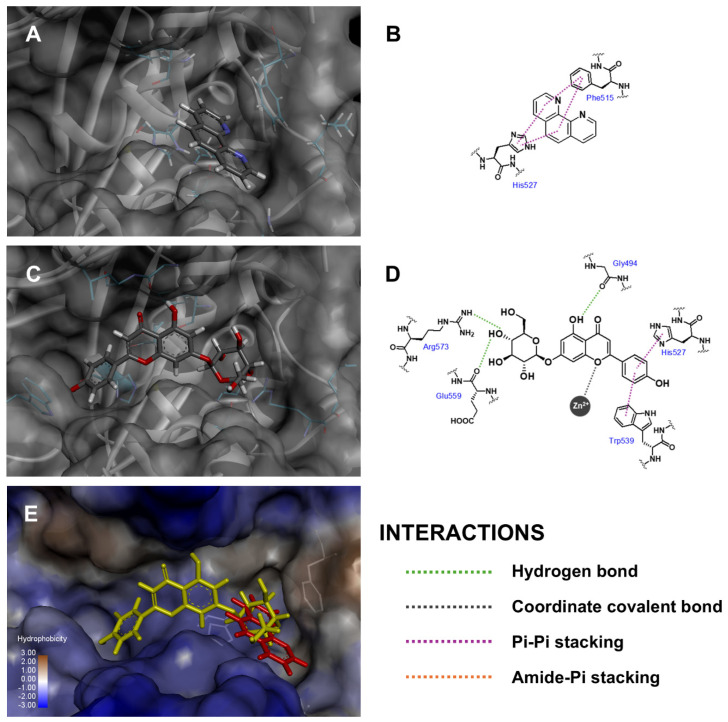
Three-dimensional conformations and two-dimensional interaction diagrams of (**A**,**B**) 1,10-phenanthroline, (**C**,**D**) Api-7-*O*-Glc in collagenase enzyme, along with (**E**) superimposed view of 1,10-phenanthroline (red) and Api-7-*O*-Glc (yellow).

Three-dimensional conformations and two-dimensional diagrams were visualized to illustrate the binding interactions between the docked molecules and the amino acid residues within the binding cavity of skin-aging-related enzymes, as summarized in Table 2. Api-7-*O*-Glc formed mutual interactions within the collagenase binding pocket as shown in Figure 3, including metal coordination with zinc (II) ion, pi–pi stacking with His527 and three hydrogen bonds with Gly494 (backbone carbonyl to 5-hydroxyl), Glu559 (backbone carbonyl to 4″-hydroxyl) and Arg573 (guanidine side chain to 4″-hydroxyl). Two critical factors influenced the fitness scores: (i) coordinated binding to zinc (II) ion, which played a crucial role as a cofactor in the enzyme’s catalytic activity [63], and (ii) the conventional hydrogen bonds, which were recognized as strong intermolecular electrostatic interactions. These interactions contributed to Api-7-*O*-Glc achieving a higher fitness score compared to 1,10-phenanthroline, which only formed pi–pi stacking with Phe515 and His523, along with van der Waal’s interactions with Glu524 and Arg573. Thus, this analysis suggested that Api-7-*O*-Glc may exhibit more potent anti-aging activity against collagenase enzymes than the positive control.

**Figure 4:**
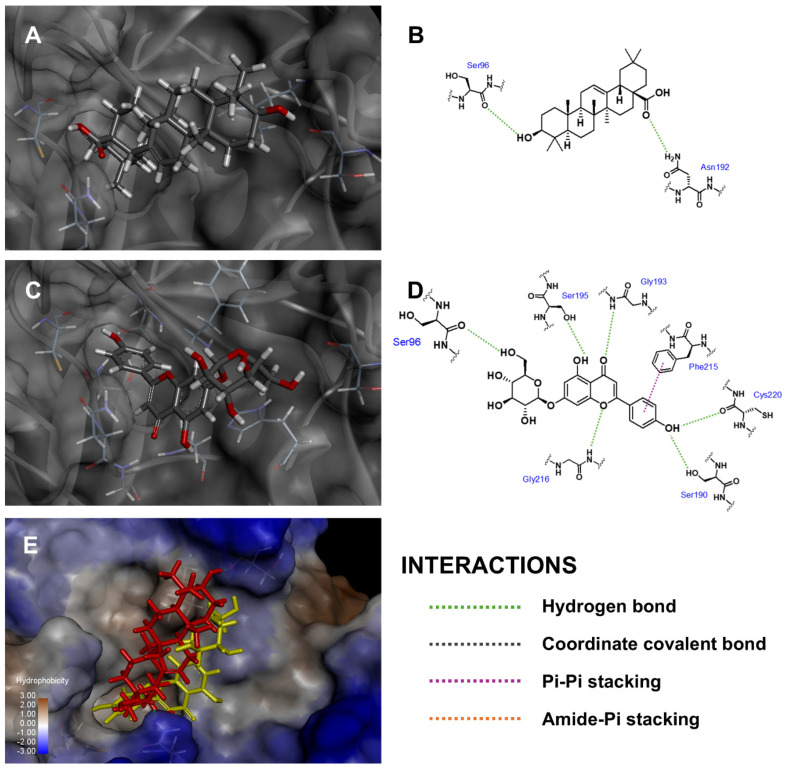
Three-dimensional conformations and two-dimensional interaction diagrams of (**A**,**B**) oleanolic acid, (**C**,**D**) Api-7-*O*-Glc in elastase enzyme, along with (**E**) superimposed view of oleanolic acid (red) and Api-7-*O*-Glc (yellow).

In the case of elastase enzyme binding site, Api-7-*O*-Glc oriented its glucoside moiety toward Ser96 and aligned similarly to oleanolic acid, the positive control, as shown in Figure 4. Api-7-*O*-Glc established six hydrogen bonds with Ser96 (carbonyl backbone to 6‴-hydroxyl), Ser190 (hydroxyl side chain to phenolic moiety), Gly193 (amide backbone to carbonyl), Ser195 (hydroxyl side chain to 5-hydroxyl), Gly216 (amide backbone to 1-oxygen atom) and Cys220 (carbonyl backbone to phenolic moiety). Additionally, it also formed one pi–pi stacking interaction with Phe215 and van der Waal’s interaction with Leu99. This alignment positioned the polar sugar moiety of Api-7-*O*-Glc next to the hydrophobic side chain of surface amino acid residues, creating some unfavorable interactions and contributing to a relatively lower fitness score. Nevertheless, Api-7-*O*-Glc still achieved a higher fitness score than oleanolic acid, which formed two hydrogen bonds with Ser96 (carbonyl backbone to 3-hydroxyl) and Asn192 (amide side chain to carboxyl). Accordingly, this result suggested that Api-7-*O*-Glc was the more effective elastase enzyme inhibitor than oleanolic acid.

**Figure 5:**
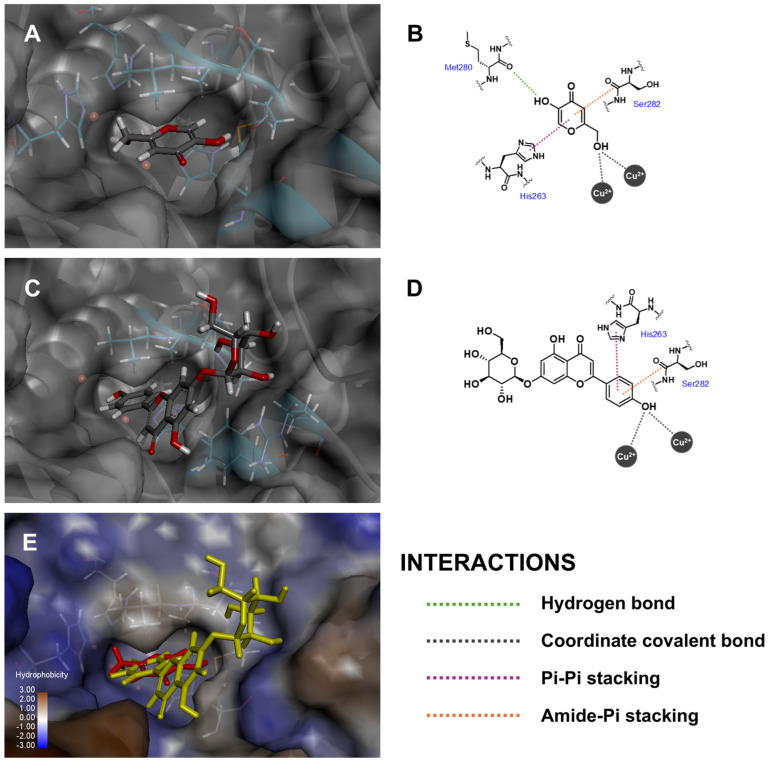
Three-dimensional conformations and two-dimensional interaction diagrams of (**A**,**B**) kojic acid, (**C**,**D**) Api-7-*O*-Glc in tyrosinase enzyme, along with (**E**) superimposed view of kojic acid (red) and Api-7-*O*-Glc (yellow).

Tyrosinase enzyme catalytic activity is categorized as an oxidation process, in which a monophenolic unit is transformed into an orthoquinone moiety via a binuclear copper (II) ion center [64]. According to the docking results, the phenolic ring C of Api-7-*O*-Glc was positioned within tyrosinase’s binding pocket, forming a coordinated covalent bond with the copper (II) ions (Figure 5), which corresponded to the enzymatic conversion of L-tyrosine to *o*-dopaquinone [65]. Additionally, Api-7-*O*-Glc formed a pi–pi stacking interaction with His263 and van der Waals interactions with His61 and His85. While no intermolecular hydrogen bonds were observed between Api-7-*O*-Glc and the tyrosinase enzyme, Api-7-*O*-Glc exhibited a silently higher fitness score than kojic acid, the positive control. Kojic acid’s interactions with tyrosinase included coordinated covalent bonds between its methanol hydroxyl group and the copper (II) ions, a hydrogen bond between the 5-hydroxyl group and the carbonyl backbone of Met280 and one pi–pi stacking interaction with His263. Although the fitness scores were not markedly different, these docking results suggested that Api-7-*O*-Glc exhibits competitive binding affinity and may serve as an effective tyrosinase inhibitor.

Although the selected crystal structures used in molecular docking experiments are not derived from human sources, they are widely utilized as initial models for evaluating anti-aging activity in both in silico and in vitro analysis [53,54,66,67,68]. Porcine pancreatic elastase has been reported to share a closely similar topology and inhibition mechanism with its human elastase [53,69,70]. While *Clostridium* collagenase and mushroom tyrosinase differ in domain compositions from their human equivalents, they belong to the same enzyme classification and perform similar functions. Both bacterial and human collagenase are metalloproteases with Zn^2+^ at their catalytic site, catalyzing the hydrolysis of collagen peptides, which contributes to reduced skin tonus, deep wrinkles and loss of resilience [71,72]. Similarly, the key enzymatic function of both fungal and human tyrosinases is melanogenesis driven by Cu^2+^-mediated metallooxidase activity. This process converts L-tyrosine to L-3,4-dihydroxyphenylalanine (L-DOPA), leading to melanin formation, which causes skin darkening and the development of freckles [67,73]. Thus, these supporting pieces of evidence provide a strongly reliable basis for the preliminary study of anti-aging activity.

### 3.2. Pharmacokinetic Properties and Toxicities

In drug discovery, pharmacodynamic properties are the initial priority factors for evaluating drug candidates; however, pharmacokinetic properties play a critical role in determining the viability of these drug candidates in terms of efficacy, safety and distribution potential [74,75]. To assess the ADMET characteristics of Api-7-*O*-Glc, three predictive webservers—SwissADME, pkCSM and ProTox 3.0—were utilized, with the results summarized in Table 3. For oral drug candidates, Lipinski’s rule of five serves as a widely accepted criterion to indicate an orally bioavailability and gastrointestinal (GI) absorption potential of lead compounds [76]. Api-7-*O*-Glc did not fully comply with Lipinski’s rule, specifically due to an excess of hydrogen bond donors. This compound was classified as a compound with lower GI absorption potential, which may limit its oral bioavailability.

The skin permeability of Api-7-*O*-Glc, defined by the Log Kp values, was calculated to be −2.735 cm/h, which exceeds the threshold of −3.0 cm/h typically associated with high skin permeability [77,78], indicating moderate skin absorption properties. Furthermore, skin sensitization predictions classified Api-7-*O*-Glc as non-irritants to skin. Toxicity predictions estimated a median lethal dose (LD_50_) exceeding 5000 mg/kg, classifying Api-7-*O*-Glc in category 5 toxicity under GHS classification [79], and showed no hepatotoxic and carcinogenic risks. These predictions support the safety profile of Api-7-*O*-Glc as an active ingredient, emphasizing its potential suitability for nutraceuticals, functional food and/or cosmeceutical applications. Moreover, its preparation as a topical formulation is likely to be more beneficial than oral supplements, based on ADMET predictions.

### 3.3. In Vitro Anti-Aging Activities

The results from in vitro aging-related enzyme inhibition assay show that Api-7-*O*-Glc, which is the most abundant major flavonoid occurring in *M. angustifolia* medicinal plants, exhibits anti-aging potential against three skin-aging-related enzymes, especially anti-collagenase and anti-elastase (Table 4). Furthermore, Api-7-*O*-Glc plays an important role as an anti-aging agent and shows a higher percentage of enzyme inhibition than the controls for collagenase (59.18 ± 6.24% of enzyme inhibition) and elastase (56.13 ± 7.49% of enzyme inhibition) (Table 4). However, this compound plays a weak anti-aging role in inhibiting tyrosinase (54.18 ± 6.54% of enzyme inhibition) compared with the controls.

On the one hand, weak anti-tyrosinase activity was also reported in the studies of Sezen Karaoğlan and his team in 2023 [80] and the team of Nasr Bouzaiene in 2016 [81], but on the other hand, the anti-aging effects of apigenin-7-*O*-glucoside on collagenase and elastase were not investigated in those previous studies. In comparison with the results from in silico assessment, which showed that apigenin-7-*O*-glucoside is able to inhibit all three enzymes, with a higher % inhibition compared with their controls, the results from in vitro assessment revealed that this flavone is the more potent anti-aging agent to inhibit collagenase and elastase (Table 4), with a higher % inhibition compared with their controls with the statistical differences. In the case of in vitro anti-tyrosinase, this compound exhibited weak anti-aging potential, with a lower % of enzyme inhibition compared with its control (Table 4). This inconsistency between the in silico molecular model and in vitro assessment commonly occurred not only in this current study but also the other previous published works [82,83,84], especially in the case of anti-tyrosinase, which the in silico results often showed a higher % of tyrosinase inhibition [82,83] than that of in vitro analysis because the in silico results are often influenced by the molecular size of the docked molecules. Reasonable binding poses and higher levels of binding interactions between docked molecules and proteins are the key factors affecting the docked score or binding affinity. While both molecules and proteins exhibit flexibility during the in vitro testing, kojic acid, a smaller molecule, may effectively utilize this flexibility more effectively than Api-7-*O*-Glc, leading to stronger interactions with the tyrosinase enzyme and a greater percentage of enzyme inhibition, like the previous works in 2025 from Tahtaci et al. [82] and Jung et al. [83]. However, the results from both in silico and in vitro models in this recent study exposed a similar trend to show that apigenin-7-*O*-glucoside is a potential anti-collagenase and anti-elastase, respectively. It is clearly seen that comparative studies using both in silico molecular docking and in vitro models provide useful fundamental data to find the most effective anti-aging property of the studied compound, which will be helpful for future research and development of phytopharmaceutical products.

Additionally, apigenin aglycone was also reported to be able to reduce the expression of collagenase [85]. In addition, the result from this current study is also consistent with many previously published works that reported on the potential of flavonoids as the anti-aging phytochemical compounds for nutraceuticals, functional food/cosmeceutical or phytopharmaceutical applications [27,80,81,86,87,88,89,90,91,92,93]. It is worth noting that these proteins were from non-human sources due to the commercial availability of these enzymes for in vitro testing. Importantly, the collagenase from *Clostridium histolyticum* and pancreatic porcine elastase has typically been used for screening various compounds/plant extracts as inhibitors [53,92]. Likewise, tyrosinase from the mushroom, *Agaricus bisporus*, is commonly employed as an enzymatic in vitro model for developing skin-whitening substances that target human tyrosinase [54,93]. Consequently, it can be supposed that the in vitro assays performed with these enzymes are reliable experiments for the fundamental step of anti-aging agent evaluation [94]. In addition, the results from this recent study were represented by working on non-human enzymes to determine the anti-aging properties of this abundant phytochemical compound found in *M. angustifolia* medicinal plants. It will also be interesting to find out in future work about its anti-aging potential on human enzymes.

It is commonly known that the skin’s extracellular matrix components are degraded by these aging enzymes, e.g., collagenase, elastase, and so on, leading to the loss of skin tone, deep wrinkles and reduced resilience [20,95,96,97]. In addition, the progression of the aging process, including the function of tyrosinase enzymes, can cause malignant melanoma and also pigmentary abnormalities, e.g., freckles or melisma [86,98]. Consequently, the phytochemical compounds and/or potential extracts which are able to inhibit the activity or processes of these aging enzymes are very helpful and interesting to the cosmeceutical, cosmetics and phytopharmaceutical industries. Interestingly, it is worth noting that this study is the first report on in silico molecular docking as well as the first completed report of Apigenin-7-*O*-glucoside, the most abundant phytochemical from Siam violet pearl medicinal plants, and on anti-aging potentials compared between anti-tyrosinase, anti-collagenase and anti-elastase effects. Remarkably, this Thai medicinal plant has been continuously consumed as a food ingredient in various dishes, a herbal tea and a traditional medicinal remedy since the ancient periods [5,24,28,29,30,31,32], which will be helpful to ensure the safety of using this medicinal plant as an alternative raw material for nutraceuticals, cosmetic, cosmeceutical and other phytopharmaceutical product development focusing on anti-aging properties.

## 4. Conclusions

To recapitulate, this study is the first report to determine the anti-aging capacities of the most abundant flavonoid phytochemical, Api-7-*O*-Glc, found in Siam violet pearl medicinal plants from Thailand using not only in silico molecular modeling but also in vitro enzymatic assessments such as anti-collagenase, anti-elastase and anti-tyrosinase activities. Furthermore, the pharmacokinetic properties based on quantitative structure–activity relationship databases, e.g., adsorption, distribution, metabolism, excretion and toxicity, also supported the safety profile of this flavone for nutraceuticals, functional food, cosmeceutical and/or phytopharmaceutical applications. Remarkedly, the present study mainly focuses on evaluating the in silico and in vitro anti-aging potential of the most abundant flavonoid from Siam violet pearl plant; however, it is also possible to discover the interesting biological actions/medicinal potential of other structurally related flavones, as well as the other less abundant flavonoids from this medicinal plant species that will be interesting and useful as the next research question for future research works. Nevertheless, the future clinical study of the safety and efficacy of the developed products formulated from this bioactive molecule/extract should be conducted before launching those products in the marketplace.

## Figures and Tables

**Table 1:** Fitness scores calculated based on GoldScore scoring function of Api-7-*O*-Glc and positive controls docked into collagenase, elastase and tyrosinase enzyme’s binding cavity.

Compound ^1^	Fitness Score		
	Collagenase	Elastase	Tyrosinase
Api-7-*O*-Glc	67.30	56.57	55.40
1,10-Phenanthroline	41.54	-	-
Oleanolic acid	-	37.04	-
Kojic acid	-	-	48.26

^1^ 1,10-Phenanthroline, oleanolic acid and kojic acid were used as positive control for collagenase, elastase and tyrosinase enzyme, respectively.

**Table 2:** Predicted intermolecular interactions of Api-7-*O*-Glc within the binding site of three anti-aging enzymes, in comparison with their positive controls.

Compound	Intermolecular Interaction				
	H-Bond	Coordinate Covalent Bond	Pi–Pi Stacking	Amide–Pi Stacking	Van der Waal’s Force
Collagenase					
Api-7-*O*-Glc	Gly494, Glu559, Arg573	Zn (II)	His527, Trp539		
1,10-Phenanthroline			Phe515, His523		Glu524, Arg573
Elastase					
Api-7-*O*-Glc	Ser96, Ser190, Gly193, Ser195, Gly216, Cys220			Phe215	Leu99
Oleanolic acid	Ser96, Asn192				
Tyrosinase					
Api-7-*O*-Glc		Cu (II) 400, 401	His263	Ser282	His61, His85
Kojic acid	Met280	Cu (II) 400, 401	His263	Ser282	

**Table 3:** Predicted pharmacokinetic and toxicological properties of Api-7-*O*-Glc.

ADMET Property	Api-7-*O*-Glc
MW (g/mol)	432.38
Rotatable bonds	4
Hydrogen bond donors	6
Hydrogen bond acceptors	10
Topological polar surface area (Å^2^)	170.05
Partition coefficient (Log P)	1.98
Solubility (Log S)	−3.78
Water solubility (mg/mL)	0.0719
GI absorption	Low
Skin permeability (Log Kp, cm/h)	−2.735
Skin sensitization	No
Hepatotoxicity	No
Carcinogenicity	Inactive
LD_50_ (mg/kg)	5000
Toxicity class	5

**Table 4:** The in vitro skin-aging-related enzyme inhibitions of Api-7-*O*-Glc.

Anti-Aging Activities	% of Enzyme Inhibition ^#^		Statistical Significance
	Api-7-*O*-Glc	Controls	
Collagenase	59.18 *±* 6.24	33.14 ± 1.84	**
Elastase	56.13 *±* 7.49	49.90 ± 1.70	*
Tyrosinase	54.18 *±* 6.54	67.90 ± 1.90	*

^#^ Kojic acid was employed as the specific inhibitor of tyrosinase, 1,10-Phenanthroline was employed as the specific inhibitor of collagenase and oleanolic acid was used as the specific inhibitor of elastase. ** significance at *p* < 0.01; * significance at *p* < 0.05.

## Data Availability

Data availability on request due to restrictions.

## Supplementary Materials

The following supporting information can be downloaded at: https://www.mdpi.com/article/10.3390/antiox14030272/s1, Table S1: Fitness score of phytochemical flavonoids isolated from *Monochoria angustifolia* (G. X. Wang) Boonkerd & Tungmunnithum docked into collagenase, elastase and tyrosinase enzymes.
