# Supplementary material for: Discovering Skin Anti-Aging Potentials of the Most Abundant Flavone Phytochemical Compound Reported in Siam Violet Pearl, a Medicinal Plant from Thailand by In Silico and In Vitro Assessments
Source: Antioxidants (Basel). 2025 Feb 26;14(3):272. doi: 10.3390/antiox14030272 (PMC11939551; doi:10.3390/antiox14030272)
Supplement: Supplementary file 1 [file antioxidants-14-00272-s001.zip › antioxidants-3420491-supplementary.pdf]

Suplementary Materials

Discovering Skin Anti-Aging Potentials of the Most Abundant Flavone Phytochemical Compound Reported in Siam Violet Pearl, a Medicinal Plant from Thailand by In Silico and In Vitro Assessments

Chaiyawat Aonsri [1,2], Sompop Kuljarusnont [3] and Duangjai Tungmunnithum [4,5,*]

1 Department of Pharmaceutical Chemistry, Faculty of Pharmacy, Mahidol University, Bangkok 10400, Thailand; chaiyawat.aon@mahidol.ac.th
2 Unit of Compounds Library for Drug Discovery, Mahidol University, Bangkok 10400, Thailand
3 Department of Obstetrics and Gynecology, Faculty of Medicine Siriraj Hospital, Mahidol University, Bangkok 10700, Thailand; sompop.kul@mahidol.edu
4 Department of Pharmaceutical Botany, Faculty of Pharmacy, Mahidol University, Bangkok 10400, Thailand
5 Le Studium Institute for Advanced Studies, 1 Rue Dupanloup, 45000 Orléans, France
* Correspondence: duangjai.tun@mahidol.ac.th; Tel./Fax: +66-26448696

Table S1. Fitness score of phytochemical flavonoids found in *Monochoria angustifolia* (G. X. Wang) Boonkerd & Tungmunnithum docked into collagenase, elastase and tyrosinase enzyme.

Compound [1]	Fitness score		
	Collagenase	Elastase	Tyrosinase
Api-7-*O*-Glc	67.30	56.57	55.40
Api	52.79	52.43	55.01
Lut-7-*O*-Glc	76.83	57.18	58.60
Lut	57.58	52.56	57.63

[1] The abbreviation of compounds are described as follows: Api = Apigenin; Lut-7-O-Glc = Luteolin-7-O-glucoside; Lut = Luteolin.
